# Supplementary material for: Distribution of the Water-Soluble Astaxanthin Binding Carotenoprotein (AstaP) in Scenedesmaceae
Source: Mar Drugs. 2021 Jun 20;19(6):349. doi: 10.3390/md19060349 (PMC8234090; doi:10.3390/md19060349)
Supplement: Supplementary file 1 [file marinedrugs-19-00349-s001.zip › marinedrugs-1246584-supplementary.pdf]

S. acutus (ACB06751): pI=3.95, no putative glycosylation site

MKLFAAVVLVTCALASVQGRTLQQAAAAPTTVAQALAQTPSLSTLNAAVQAAGIDIPADAAW***TIF***
APKNEAFSDDDIREETGLTAQQLLQPENKQALTQLLQYHIVPSGALRAAQLQDGQQLTTALAGAA
PLKVDIEDEKVEIEVPDAGNDDDGDDADVEQADMTVG***NSVIHIVDEVL***IPASLRGSRQPARSG

C. vacuolata (CvacAstaP1 MZ063685): pI=6.12, 10 putative glycosylation sites

MRTLALFVLACTIGAAYGRELLQAAPAASYNTLAQAVAAANASAPNLSILLAAVKAANVSGALGP
NTTW***TILAPTNQAF***TSRLNESLGITPQQLLQPANKKTLVEVLSYHVIPSGAVYSSNLTNGQEVPT
ALAGADPLTVAINNRTVTFEGAENNATVTVANIRAG***RSVIHVINDVL***LPAGVGNSSNTTAEGEST
APGAAGSRDVAAGSAVGGGSTSAPAPAAAKGAAGAAQVPAVLLASMLLIGMLF

C. vacuolata (CvacAstaP2 MZ063686): pI=10.3, 2 putative glycosylation sites

MARNAIVFLAIVCLVAGATADKADPAKKKSAPSYPSLSDAVKAAATAPQLTTLLAAINAAGVASA
LKPDTAW***TILAPTNKAF***ETRLAKLNVTADALLKNKDLLVKVLSYHVIPSGAVYSSQLKDKQTVAT
ALKNATLTVKIYGKNVAFKGPVNKAKVVVADIKAG***KSVVHVIDDVL***VPPGVVSPEVAQKWKAEYK
SKKDASMKKMDKP

Crl. variabilis (EFN56922): pI=4.15, 1 putative glycosylation site

MARHSAQSYDNIGELLDDTPKLSILKAALEAAGLDELASDDALEA***TLFAPTDKAF***ADAITALNLT
AEEVLGDKDLLTTVLSYHVIPGVAVEAAALEEGQILQTILEGAAGQLKYYTFKGLPRLRTTADQV
ARITETDISAG***KAIVHLIDAVL***IPGDEFFSAPAPGPSP

Cmy. reinhardtii (XP001700577): pI=6.33, 15 putative glycosylation sites

MARWTRPGASTILVASCLLLAVAGSSAQTILQAIQSANLTLLAAGLQVADLATTLNSTTGPNITLF
APSDDALLAMTAALNTTPNALLTLGPKVAPVFTYHAITTPLLAADIPTGDTPYATLNTGLNLTVSK
AANGSVVVRALGSDANVIRTDITAGGAVVHVIDSVLLPFFLSVASAATRTPQLSSLLSAVASNGLA
PALSNPNLNV***TVFAPVNDAF***NASSAYLTSVNASITDVLTYHVATSRVLNGSEVTASPITLTTLNSR
ANLTVARDGNNVVVTPVGATAATVLAANIPVGLDLTTGGP***RTFVHLVNAVL***LPFYTTVANAAERAG
LTTLVAAVAASDPAFLAAVTDPTFRG***TILAPSNAAF***AATLASLNVTAAQLLADKDNLRRILNAHII
PNAAVFSNQLSNNQTVNTTGGALLTVSIGNGIVRFNAAKSSAKVVAADVSIGNGRAVVHVIDRVLL
PADLTLPTPYVTVAGAAQAANLSTLLAAVTASDASFLTTLTDPDFNGTVLAPTPLSDCHVALLMPN
AAFTAALTALGVNATQLLADKDNLRRILNAHIITPGAVRSSQLVNNQNITTLDGVLTVRINGTGVF
FVAAKSTAKVVTPDVIAG***NAVVHTIDFVL***LPASVTLTTGGSGGGGSGGGGAAGMASPSVLTLLSSA
LAFLALFAFGRF

Figure S1. The deduced amino acid sequences of the AstaP orthologs. N-terminal signal peptides are highlighted by yellow. H1 and H2 motifs of the fasciclin domains are shown in boldface italics. Potential sites for N-linked glycosylation are shown in red fonts.

```
CvacAstaP1      -------------------------------MRTLALFVLACTIG--AAYGRELLQAAPA 27
CvacAstaP2      MGRPCLLTYTQTQSYLIDFVVTPTSQQSAMARNAIVFLAIVCLVAGATADKADPAKKKSA 60
                                                .::.::.:.* :.  :*   :  :  .*

CvacAstaP1      ASYNTLAQAVAAANASAPNLSILLAAVKAANVSGALGPNTTWTILAPTNQAFTSRLNESL 87
CvacAstaP2      PSYPSLSDAVKAA-ATAPQLTTLLAAINAAGVASALKPDTAWTILAPTNKAFETRLAK-L 118
                .** :*::** ** *:**:*: ****::**.*:.** *:*:********:** :** : *

CvacAstaP1      GITPQQLLQPANKKTLVEVLSYHVIPSGAVYSSNLTNGQEVPTALAGADPLTVAINNRTV 147
CvacAstaP2      NVTADALLK--NKDLLVKVLSYHVIPSGAVYSSQLKDKQTVATALKNAT-LTVKIYGKNV 175
                .:*.: **:  **. **:***************:*.: * *.*** .*  *** * .:.*

CvacAstaP1      TFEGAENNATVTVANIRAGRSVIHVINDVLLPAGVGNSSNTTAEGESTAPGAAGSRDVAA 207
CvacAstaP2      AFKGPVNKAKVVVADIKAGKSVVHVIDDVLVPPGV------------VSPEVAQKWKAEY 223
                :*:*. *:*.*.**:*:**:**:***:***:*.**            .:* .* . ..

CvacAstaP1      GSAVGGGSTSAPAPAAAKGAAGAAQVPAVLLASMLLIGMLF 248
CvacAstaP2      KSKKDASMKKMDKP--------------------------- 237
                 *  ... ..   *
```

Figure S2. Comparison of amino acid sequences among AstaP-proteins in *C. vacuolata*. Amino acid sequence similarity between CvacAstaP1 and CvacAstaP2 showed 44% identity in overlapping regions.
